# Strengthening mental health systems in low- and middle-income countries: the Emerald programme

**DOI:** 10.1186/s12916-015-0309-4

**Published:** 2015-04-10

**Authors:** Maya Semrau, Sara Evans-Lacko, Atalay Alem, Jose Luis Ayuso-Mateos, Dan Chisholm, Oye Gureje, Charlotte Hanlon, Mark Jordans, Fred Kigozi, Heidi Lempp, Crick Lund, Inge Petersen, Rahul Shidhaye, Graham Thornicroft

**Affiliations:** King’s College London, Institute of Psychiatry, Psychology and Neuroscience, 16 De Crespigny Park, London, SE5 8AF UK; Addis Ababa University, College of Health Sciences, School of Medicine, Department of Psychiatry, 6th Floor, College of Health Sciences Building, Tikur Anbessa Hospital, PO 9086, Addis Ababa, Ethiopia; Department of Psychiatry, Universidad Autonoma de Madrid, IIS-IP, CIBER, Calle Arzobispo Morcillo 4, 28029 Madrid, Spain; Department of Mental Health and Substance Abuse, World Health Organization, Avenue Appia 20, 1211 Geneva 27, Switzerland; Department of Psychiatry, College of Medicine, University of Ibadan, University College Hospital, Oritamefa, PMB 5116, Ibadan, Nigeria; Department of Research and Development, HealthNet TPO, Lizzy Ansinghstraat 163, 1072 RG Amsterdam, Netherlands; Butabika National Referral and Teaching Mental Hospital, PO Box 7017, Kampala, Uganda; King’s College London, Faculty of Life Sciences and Medicine, Academic Rheumatology, Clinical Trials Group, Weston Education Centre, 10, Cutcombe Rd., London, SE5 9RJ UK; Alan J Flisher Centre for Public Mental Health, Department of Psychiatry and Mental Health, University of Cape Town, 46 Sawkins Road, Rondebosch, 7700 Cape Town, South Africa; School of Applied Human Sciences, University of KwaZulu-Natal, Mazisi Kunene Avenue, 4001 Durban, South Africa; Center for Chronic Conditions and Injuries, Public Health Foundation of India, Plot No. 47, Sector 44, Institutional Area Gurgaon, Delhi, NCR 122002 India

**Keywords:** Delivery of health care, Health care systems, Mental health

## Abstract

There is a large treatment gap for mental health care in low- and middle-income countries (LMICs), with the majority of people with mental, neurological, and substance use (MNS) disorders receiving no or inadequate care. Health system factors are known to play a crucial role in determining the coverage and effectiveness of health service interventions, but the study of mental health systems in LMICs has been neglected. The ‘Emerging mental health systems in LMICs’ (Emerald) programme aims to improve outcomes of people with MNS disorders in six LMICs (Ethiopia, India, Nepal, Nigeria, South Africa, and Uganda) by generating evidence and capacity to enhance health system performance in delivering mental health care. A mixed-methods approach is being applied to generate evidence on: adequate, fair, and sustainable resourcing for mental health (health system inputs); integrated provision of mental health services (health system processes); and improved coverage and goal attainment in mental health (health system outputs). Emerald has a strong focus on capacity-building of researchers, policymakers, and planners, and on increasing service user and caregiver involvement to support mental health systems strengthening. Emerald also addresses stigma and discrimination as one of the key barriers for access to and successful delivery of mental health services.

## Background

A health system can be defined as “*the sum total of all the organizations, institutions, and resources whose primary purpose is to improve health*” [1]. A well-functioning health system should deliver services of adequate quality to all people, whenever and wherever they need them [1], and should protect the right to health for everyone, including people with mental, neurological, and substance use (MNS) disorders [2,3], whether through professional services or non-professional care services such as family or self-care.

However, health systems often fail to meet the needs of people with MNS disorders. They are particularly overstretched in low- and middle-income countries (LMICs), due to the higher overall burden of disease in these populations compared to high-income countries and the lower availability of human and financial resources. Even though three-quarters of the global disease burden that is due to MNS disorders affects LMICs [4], and 8.9% of the disease burden in LMICs is due to MNS disorders (30.1% when excluding mortality) [5], only a very small proportion of the health budget in LMICs is allocated to the treatment and prevention of these disorders (an average of 1.9% in lower-middle income countries, and 0.5% in low-income countries) [6].

The result of this imbalance is a substantial treatment gap whereby only a small minority of people with MNS disorders receive any form of treatment, and an even smaller proportion receive appropriate and evidence-based care, i.e., care that is continuous, coordinated, and multi-sectorial. A large multi-country survey showed that, on average, 76% to 85% of people with severe mental disorders in low-income countries had not received any treatment in the previous 12 months [7]. This lack of treatment is associated with considerable consequences, including disability [7-9] and suicide [10,11].

### Recent advances in global mental health

There have been several landmark international achievements and publications that have significantly improved the knowledge base to mitigate against the substantial burden of MNS disorders. These include the World Health Report in 2001 [12]; the two Lancet series on global mental health in 2007 and 2011; the Movement for Global Mental Health [13]; WHO’s Mental Health Gap Action Programme (mhGAP) for scaling up services for MNS disorders [14,15]; a review of Grand Challenges in Global Mental Health [16]; the establishment of Collaborative Hubs for International Research in Mental Health by the National Institute of Mental Health, USA [17]; the WHO resolution in 2012 and action plan in 2013 [18] to address the global burden of MNS disorders, whose key objectives strongly reflect a health systems approach; as well as the on-going PRogramme for Improving Mental health carE (PRIME) [19,20], which aims to develop, deliver, scale-up, and evaluate evidence-based packages of care in five African and Asian countries.

However, most of the existing knowledge base and on-going work is focused on the prevalence of MNS disorders, and evidence of effectiveness and feasibility of local interventions, with particular emphasis on the adoption of task-sharing to increase access to integrated services. What is still lacking is proof and capacity in mental health system strengthening, i.e., the health system requirements necessary to scale-up integration of mental health care into other health systems (particularly primary health care) in LMICs. This includes health system inputs (for instance, human and financial resource development), health-system processes, and system-level information outputs, as well as knowledge exchange and dissemination. This is especially important for LMICs, which are often undergoing an epidemiological transition of disease from infectious or communicable diseases towards a rising burden of chronic illnesses, including non-communicable conditions such as MNS disorders.

It is imperative that health systems adapt to provide the collaborative (integrated) model of care shown to best meet the needs of people with chronic disorders [21]. Often in LMICs the existing health systems are more orientated to acute conditions, which results in fragmented care, erratic medication supplies, resource problems, or lack of sustainability of services for long-term disorders. It is these issues at the health-system level that the Emerald programme is committed to address.

## Aims and objectives of Emerald

Emerald is an international programme of work running from 2012 to 2017 [22]. The aims are to improve mental health outcomes in six LMICs by generating evidence and capacity to enhance health system performance, thereby improving mental health care in the respective countries, and helping to reduce the mental health treatment gap. It aims to do so by i) identifying key barriers within health systems to the effective delivery of mental health services, and ii) offering solutions for an improved delivery of mental health services in the future.

**Figure 1:**
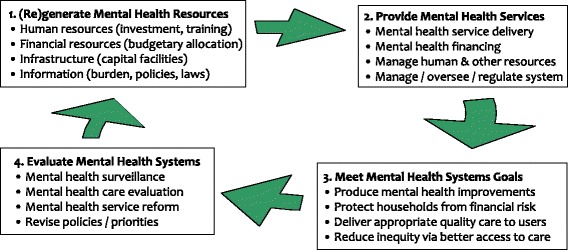
**Conceptual scheme linking system inputs, processes, and outputs in the Emerald programme.**

Figure 1 shows a conceptual schema of the key aspects of a mental health system. The system requires inputs (for example, human and financial resources), which can be employed to finance and deliver appropriate services. These actions produce the outputs and outcomes that the system sets for itself (including good service quality and financial protection, as well as improved health). System inputs, processes, outputs, and outcomes are evaluated and adapted to reflect the changing needs of the population and engender improvements in the mental health system [23]. In line with this framework and the goals of the WHO’s Global Action Plan for mental health (2013–2020) [18], the three overarching objectives of Emerald are to address: i) adequate, fair, and sustainable resourcing for mental health (health system inputs); ii) integrated provision of mental health services (health system processes); and iii) improved coverage and goal attainment in mental health (health system outputs).

In addition to these three health system objectives, fundamental to Emerald is the enhancement of in-country capacities and skills to plan, implement, evaluate, and sustain system improvements.

The programme is closely linked to, and complements, the PRIME programme [19,20]. Whilst PRIME focuses on mental health service development at the community, facility, and district level, Emerald concentrates on establishing or strengthening the mental health systems required to implement these services, particularly at the district, regional, and national levels.

## Emerald countries and sites

The Emerald programme is working to strengthen mental health systems in Ethiopia, India, Nepal, Nigeria, South Africa, and Uganda (Table 1). These countries, to differing degrees, all face mental health system challenges that are common across LMICs such as weak governance, low resource bases, or poor information systems. The six countries were invited into the programme due to the commitment of local researchers and policymakers, and the timeliness of the programme within countries (for example, relating to mental health policy or service development). Due to the diversity of the sites, for instance, with regard to their geographical, economic, socio-cultural, and urban/rural contexts, this may increase the programme’s relevance to a range of other LMIC settings.

**Table 1:** **Indicators of development, health resources, and the mental health system in the six Emerald countries**

	**Ethiopia**	**India**	**Nepal**	**Nigeria**	**South Africa**	**Uganda**
**Administrative Health Units (AHU) in which Emerald is implemented**	Sodo	Sehore (Madhya Pradesh)	Chitwan	Oshogbo	Kenneth Kuanda Dist. NW Province	Kamuli
**Population of AHU**	165,000	1,311,008	575,058	288,455	632,790	740,700
***Country-level indicators***						
**Economic and financial**						
World Bank resource category	Low	Lower-middle	Low	Lower-middle	Upper-middle	Low
% GDP spent on health	5.9	4.2 ^♦^	5.3 ^♦♦^	5.0	8.4	7.3
% Health budget spent on mental health	Not known	0.06 ^♦^	0.17 ^♦♦^	0.40	4.50	0.44
**Service availability (per 100,000)**						
Mental health outpatient facilities	0.06	0.33 ^♦^	0.08 ^♦^	0.03	6.85	0.08
Psychiatric beds in general hospitals	0.04	0.82 ^♦^	1.0 ^♦♦^	0.20	2.70	1.24
Beds in mental hospitals	0.35	1.47 ^♦^	0.20 ^♦♦^	2.53	19.50	1.48
**Human resources (per 100,000)**						
Psychiatrists	0.04	0.30 ^♦^	0.13 ^♦♦^	0.12	0.27	0.09
Nurses	0.59	0.17 ^♦^	0.27 ^♦♦^	0.60	9.72	0.76
Psychologists	0.02	0.05 ^♦^	0.02 ^♦♦^	0.02	0.31	0.02
**Governance**						
Mental health policy and/or legislation that is up-to-date (i.e., updated in last 10 years) and in accordance with international human rights	Yes (policy) No (legislation)	No	No	Yes	Yes	No
**Workforce capacity and training**						
Most primary health care doctors had mental health training in last 5 years	No	No	No	No	Not known	Yes
Primary care nurses can independently diagnose and treat mental disorders	No	No	No	Yes	No	Yes
**Information systems**						
Data on number of outpatients with mental disorders	Not known	No	Yes	No	No	Yes
Data on number of persons with mental disorders treated in primary health care	Yes	No	No	No	Yes	Yes

^♦^ Data taken from WHO’s Mental Health Atlas (2011) [24].

^♦♦^ Data taken from WHO’s AIMS (2006) [25].

## Activities and methods

Emerald entails a large programme of work that is being implemented through a range of innovative methodologies (see, for example, the OneHealth tool mentioned below). In addition, emphasis is placed on service user and carer involvement, reduction of stigma and discrimination, and dissemination of research findings. To ensure the comparability and generalizability of findings, broadly the same activities and methods are employed across all six participating countries of the programme, though some country-specific adaptations may be made for data collection methods or research instruments to ensure that these are in line with the different in-country contexts and to account for the relative strengths and weaknesses of the health systems of individual countries. For instance, in investigating how to strengthen governance processes to facilitate integrated services through key informant interviews, a generic interview schedule that covered the key governance issues to be explored was initially developed; countries then adapted the schedule to ensure that it was contextually relevant. South Africa’s adaptations included, for example, ensuring that the challenges associated with implementation of the recent Mental Health Policy and Action Plan at provincial and district level were explored. India adopted the schedule to incorporate questions related to the draft Mental Health Care Bill and new National Mental Health Policy. In South Africa also, a range of local service and epidemiological data were used to adapt the OneHealth tool (see below) to the South African context. Furthermore, in Nepal, where the provision of psychotropic drugs in primary health care is largely absent, a qualitative study was conducted to better understand the barriers around procurement and distribution. In Ethiopia, the non-availability of electricity for most homes and the lack of diversity of possessions had to be taken into account when adapting the household economic survey (see below for details).

All data from the different sub-studies of the programme are analysed both on a country-specific level as well as on a cross-country level. The current status of work varies between the different sub-studies of the programme in line with the aims and objectives of the programme; whilst some are close to completion, others are ongoing or yet to commence. A case study of some of the work that is being conducted in one of the Emerald countries, Ethiopia, is provided in Box 1.

### Health system inputs

One of Emerald’s key objectives involves the identification of health system resources, finance mechanisms, and information needed to scale-up mental health services and move towards universal coverage. This is laid out across three tasks:

i). *Adequacy of resourcing for mental health*: For this, work is in progress to develop and integrate a module on MNS disorders within the United Nation’s OneHealth systems planning tool [26]. OneHealth is a tool to strengthen health system analysis, costing and financing scenarios at the country level. It does so by bringing together disease-specific planning and health systems planning, as well as incorporating modules to estimate the predicted health impact of scaling up interventions over time and for assessing fiscal space/financial sustainability. Through application of this tool, Emerald provides new estimates of the cost and impact of scaling up interventions for MNS disorders, as well as assessing the health system implications of planned scale-up. This facilitates an integration of mental health programme-specific strategies into broader national health plans. By drawing on data from the real world settings of six diverse LMICs, key requirements for and constraints to local mental health service provision and scale-up are being built into the tool regarding local mental health service provision and needs, in a manner that has not previously been possible. These include, for example, human resource availability and capacity at the primary care level, capacity to deliver psychosocial interventions, and medication availability at different levels in the system. Three capacity-building workshops in use of the OneHealth tool have already been conducted (in Ethiopia, India, and Nigeria), and currently the estimates of costs and impacts of scaling up in the six participating Emerald countries are being finalized within the OneHealth tool.
ii). *Fair financing and improved economic outcomes for mental health:* Work is underway for a large survey in each of the six participating countries with household members of people with MNS disorders who attend health care facilities in the study district (Table 1), to assess the economic impact of people living with an MNS disorder and the economic impact of improved care. The household survey is based on the previously validated WHO survey on health and ageing (SAGE) developed specifically for use in LMICs [27], but has been adapted to fit the aims and objectives of the Emerald programme. The survey includes questions around household composition, income, and spending (on health care, including sources and sectors beyond the professional such as use of traditional/religious healers, as well as other services and goods).
iii). *Sustainable financing for mental health:* This will involve data analysis as well as in-depth consultations with policymakers, planners, economists, and other stakeholders regarding potential financing mechanisms for mental health care in each country, building on findings derived from the OneHealth tool (resource needs) and the household survey (financial burden and equity).

### Health system processes

Another key objective for Emerald is the evaluation of the context, process, experience, and health system implications of mental health service implementation. All six participating countries are using local adaptations of the WHO mhGAP Intervention Guide (mhGAP-IG) [14,15] to facilitate the scaling-up of integrated mental health services. The mhGAP-IG includes diagnostic and treatment guidelines for nine MNS disorders common in LMICs, or which have a major public health impact or are associated with human rights abuses. Key strategies to support the development and implementation of mental health plans in LMICs from the district through to national levels are identified within Emerald. This is achieved, *inter alia*, through:

i). Documentary analyses of key legislation and policy documents at national, provincial, and/or district level at the beginning of the programme, to facilitate the implementation of legislative and policy imperatives (completed).
ii). Using a governance framework proposed by Siddiqi et al. [28], qualitative key informant interviews with relevant groups (such as policymakers, managers, district service providers, community service officers, service users, and carers) are being conducted at the start and end of the programme to better understand governance processes that enable or inhibit the development and implementation of mental health policies, plans, and legislature for integrated mental health care (including factors outside of the professional health care system (such as traditional/religious healers) due to the plurality of services), and to identify strategies to strengthen these processes.
iii). A mixed-method baseline and endline assessment of the impact of integrated care on the health system in the six participating countries, using questionnaires, observations within health care facilities, and semi-structured interviews with key informants.

### Health system outputs

Emerald’s third key objective focuses on the development, use, and monitoring of indicators for mental health service coverage and system performance. This is achieved by: i) review of existing information systems (completed); ii) a Delphi study, with an expert panel consisting of 93 mental health researchers, clinicians, and policymakers almost all working and residing in LMICs, who have generated and ranked a set of 52 indicators for routine measurement of mental health service coverage and system performance (ongoing); iii) in-depth interviews and focus group discussions with selected health information personnel and health care providers, to assess barriers related to the introduction and the use of selected indicators (ongoing); and iv) monitoring and evaluation of the performance and utility of the selected indicators (ongoing).

### Capacity-building in mental health systems research

In addition to the above three key objectives, Emerald has a strong focus to build up the capacity of i) local researchers, ii) policymakers and planners to implement system improvements for mental health care services, and iii) service users and caregivers in each participating country. This is realised through tailored capacity-building interventions for each of the three stakeholder groups (researchers, policymakers and planners, and service users and caregivers) that can be delivered independently within each of the Emerald countries. Approaches include ‘Training of Trainers’ courses; funding for PhD (five so far; four are still planned) and Masters students (one so far; another is planned); supervision and monitoring of PhD students; mentoring mid-level researchers; workshops and policy dialogues; advocacy and empowerment workshops for service users and caregivers; and capacity-building amongst health care providers to work towards greater service user involvement.

In addition, three Masters-level teaching modules with 28 sub-modules (Table 2) have been developed to build capacity in mental health systems research within Emerald countries and beyond, through integration of the modules into ongoing Masters courses within countries. Each of the 28 sub-modules encompasses at least one full day of face-to-face teaching, which were identified and agreed within the Emerald consortium based on the group’s expertise. The sub-modules were developed through a collaborative effort by all members of the Emerald team in the first half of 2014, both by adapting materials that had been previously developed by them or their colleagues, and by newly developing materials. A peer-review system is being employed to improve training materials, which will be freely and publicly available to use by the end of the programme.

**Table 2:** **Masters-level modules in mental health system strengthening developed within Emerald**

**Module 1: Mental health system components**	**Module 2: Mental health systems research methods**	**Module 3: Mental health system contexts – Areas of special attention**
1.1 Introduction to mental and neurological disorders	2.1 Mental health epidemiology	3.1 Stigma and discrimination
1.2 Health systems concepts and approaches	2.2 Methods to evaluate mental health interventions	3.2 Child and adolescent mental health
1.3 Mental health policy	2.3 Economic evaluation	3.3 Older adults
1.4 Leadership and governance	2.4 Qualitative research methods	3.4 Suicidal behaviour
1.5 Service organization	2.5 Collaborative care in mental health	3.5 Systems research in humanitarian settings
1.6 Promotion and prevention	2.6 Service user and action research	3.6 Women/maternal/gender issues
1.7 Health systems financing	2.7 Research ethics	3.7 Culture and mental health
1.8 Human resources	2.8 Implementation science	
1.9 Information systems and monitoring and evaluation	2.9 Knowledge translation	
1.10 Interventions and technologies, delivery systems, and essential treatments	2.10 Survival skills for researchers	
1.11 Human rights/equity		

### Service user involvement and reduction of stigma and discrimination

Partnerships with service users are essential for the development of evidence-based care in government guidance across the globe [29-31]. They may protect those who receive involuntary treatment abuses, or those who are marginalized due to their low socio-economic status or social stigma attached to MNS disorders, through their greater involvement in the implementation of mental health system processes. Close collaborations between service users/caregivers and healthcare professionals have been pioneered in mental health and HIV/AIDS worldwide, and the evidence of its usefulness is slowly emerging through a number of recent publications [32]. Service users and their families and caregivers are thus involved in all components of the Emerald programme, for example, through consultations, including qualitative work, to better understand contextual factors, capacity-building, and advocacy activities, and to pilot collaboration to embrace involvement of all stakeholders.

Since the quantity and level of involvement of service user organizations varies widely between Emerald countries (for example, in Uganda, 16,900 service users are members of service user organizations, whereas in Ethiopia there are no such organizations), country-specific strategies are being employed. As part of this, stigma and discrimination are addressed as one of the key barriers for access to and successful delivery of mental health services in LMICs [33-35]. This involves a two-way process, in which increased service user and caregiver involvement is established within the programme, and lessons are garnered on how best to reduce stigma through interviews with service users and caregivers.

### Dissemination

The Emerald programme is working to disseminate its research findings widely to engage with different stakeholder groups (such as Ministries of Health and Finance in study countries, policymakers and planners, national and international development agencies, non-governmental organizations working in mental health, mental health researchers, service users and providers, and caregivers). This includes the establishment of mental health research networks within the programme and beyond. Channels that are employed for this are joint publications in scientific journals, policy briefing papers, conference presentations and posters, a project website, project flyer, social media sites, and press conferences.

## Challenges

For Emerald, there are several challenges that are specifically addressed through each of the programme’s objectives as outlined above. These include inadequate resources for mental health, limited finances, poorly trained staff, a lack of understanding about service delivery processes and quality improvement, poor outcome assessment through health management information systems (HMIS) (for example, in India, due to a lack of a robust monitoring framework and the non-integration of mental health indicators with HMIS), difficulties in exchange of knowledge, and in some countries the low level of empowerment and the marginalisation of service users and caregivers (in Ethiopia, for instance).

One of the main barriers is the translation of the programme’s findings into practice, particularly to actively involve decision-makers in the six participating countries to bring about changes in mental health policy and systems strengthening for integrated mental health service provision. For example, in Nepal, the high turn-over of staff at senior policy levels creates barriers for mental health system strengthening in terms of having a solid group of policymakers to advocate and work with. In India, poor community participation and ownership of the mental health programme form similar barriers. To address this, but also to improve the applicability of the programme within each of the participating countries, links and partnerships with policymakers, planners, and other stakeholder groups have been established early on in the programme. Indeed, an important strength of Emerald is the direct involvement of key policymakers from the Ministries of Health in the six countries as partners who have been actively engaged from the very inception of the programme and who contribute to the implementation of Emerald throughout its tenure.

## Building sustainability

The Emerald programme seeks to strengthen mental health systems in six LMICs by working on health system inputs, system processes, and performance outputs that are related to mental health service delivery, thereby addressing a key implementation science gap. Based on the experience of the participating countries, the programme aims to produce a research-informed ‘roadmap’ for decision-makers in LMICs on how best to scale-up mental health services within the constraints of the broader health system, including the identification of the human and budgetary resource needs to meet locally-determined targets, health financing policy options, governance requirements, and coverage/performance indicators. Furthermore, Emerald aims to map out and articulate the pathways used in the six local health systems to integrate mental health care within existing services. Through documentation of the impact of this integration, the programme offers health service providers, both in the six countries and beyond, workable and tested strategies for sustainable integration. Another major impact of this programme is the identification, training, and support for the career progression of mental health professionals and researchers in LMICs with the information and skills needed to bring a health systems perspective to mental health planning, provision, and evaluation – one that complements existing knowledge, capacities, and learning opportunities. Indeed, the shortage of technical know-how has been identified as a major barrier to the scale-up of mental health services in LMICs, and Emerald aims to address this. With this comprehensive approach, we plan to improve the evidence base on how to enhance health system performance and build capacity to support scaling-up of integrated mental health care in practice in LMICs.

## Box 1 Example case study of the Emerald programme in Ethiopia

Ethiopia’s Federal Ministry of Health is confronting a mental health care gap (i.e., the number of people with severe mental disorder who receive no treatment) of over 90% for people with severe mental illness. In response, the Ministry has launched ambitious plans to scale-up mental health care integrated into primary care services in line with the WHO’s Mental Health Gap Action Programme. A National Mental Health Symposium was convened in August 2014 to bring together key stakeholders and galvanise support for the scale-up. In support of these efforts, timely information is being provided by the Ethiopia Emerald programme’s qualitative study with national and district-level health service planners, which identified key system barriers (e.g., weak systems for monitoring, evaluating, and learning as scale-up proceeds) and facilitators to scale-up (e.g., high level political will). A workshop will be held to feed back the findings to health care planners and generate dialogue about a framework for intervention to address system barriers. Also drawing on these findings, short courses have been developed by Emerald that will seek to build the capacity of healthcare planners to strengthen mental health systems in Ethiopia. In synergy with these efforts, the Emerald-supported adaptation of the OneHealth tool has already been employed for mental health care planning for the next 5-year cycle by the Ministry of Health.

## Abbreviations

Emerald: Emerging mental health systems in low- and middle-income countries
HMIS: Health management information systems
LMICs: Low- and middle-income countries
mhGAP: WHO Mental Health Gap Action Programme
mhGAP-IG: WHO mhGAP Intervention Guide
MNS: Mental, neurological and substance use
PRIME: PRogramme for Improving Mental health carE
WHO: World Health Organization
